# Vestibular Rehabilitation Considerations in an Uncommon Optic Neuritis: A Case Report

**DOI:** 10.7759/cureus.53423

**Published:** 2024-02-01

**Authors:** Mansee S Dangare, Anam R Sasun, Pallavi Harjpal

**Affiliations:** 1 Neurophysiotherapy, Ravi Nair Physiotherapy College, Datta Meghe Institute of Higher Education and Research, Wardha, IND

**Keywords:** neurophysiotherapy, physiotherapy rehabilitation, gaze stability exercises, ‏strabismus, optic neuritis

## Abstract

Optic neuritis is an inflammatory condition that leads to inflammation and damage to the optic nerve, causing visual disturbances and pain. It is commonly associated with disorders such as multiple sclerosis and often manifests as sudden, unilateral loss of vision or blurred vision. This disorder can affect individuals of any age and may lead to decreased binocular vision, potentially resulting in difficulties with depth perception and visual coordination. Physiotherapy plays a crucial role in treating optic neuritis by addressing various aspects of the illness. We report the case of a 14-year-old male with diminution in both eyes, which was sudden in onset and painless in nature, with no history of falls, trauma, or diabetes. Magnetic resonance imaging reveals hyperintensity on short-tau inversion recovery (STIR) with mild contrast enhancement in the posterior aspects of the bilateral optic nerves (intracranial part), extending to the optic chiasm in optic neuritis. Physiotherapists employ a range of techniques to enhance the patient's overall well-being, including gaze stability exercises, eye-hand coordination exercises, and habituation exercises aimed at improving visual tracking and coordination. Additionally, physiotherapy can help reduce related symptoms such as muscle weakness, balance issues, and posture problems caused by impaired visual perception. Physical therapists endeavor to improve the quality of life for patients with optic neuritis by enhancing functional independence and contributing to a more effective approach to treatment. Notably, there was an improvement in visual scanning, spatial awareness, and eye movement control in this case.

## Introduction

The most prevalent reason for optic neuritis (ON) in both children and adults is swelling along the course of the optic nerve. The prognosis and treatment of ON will differ based on the etiology, duration, and degree of visual loss, antecedent damage, and the efficacy of previous therapy. Optic nerve inflammation can be caused by autoimmunity, infection, granulomatous disease, paraneoplastic disorders, or demyelination. Prompt identification of the cause of ON is crucial to provide fast and efficient treatment [1]. ON is characterized by visual acuity loss, visual field loss, color vision abnormalities, and an afferent pupillary defect in the affected eye. Unless a person has bilateral disease or a history of optic nerve damage in the other eye, the absence of an afferent pupillary defect should always raise concerns about the diagnosis [2]. ON is classified into four types according to the area of involvement: retrobulbar neuritis (2/3 of cases) with normal optic disc structure; papillitis with disc swelling; perineuritis affecting the optic nerve sheath while the optic disc is either normal or swollen; and neuroretinitis involving edema of the optic disc and exudating in the macular star [3,4]. The ability to see well is essential for equilibrium management. As a component of this integrated sensory feedback system, it can sustain bipedal upright stabilization throughout locomotion [5]. As an outcome of this sensory conflict, the body misaligns in a certain orientation, resulting in unequal weight-bearing, and these imbalances lead to balance problems [6]. Visual movement issues may also have a wide-ranging influence on daily tasks and freedom by making it harder to maintain the correct position and adequate eye movement [7]. Strabismus, often known as squint, is any misalignment of the optical axis. While strabismus is sometimes referred to as a lazy eye, the term is used to describe a variety of conditions, including amblyopia (low vision in an eye without signs of physical deformity or disease), ptosis, and strabismus itself. Strabismus affects between 1% and 3% of children [8]. Children with a history of premature birth, systemic illnesses like cerebral palsy, genetic disorders, and a family history of strabismus are more likely to experience it [9]. Strabismus most frequently manifests on the horizontal axis. An exotropia is an outward drift of one eye, while an esotropia is the inward crossing of one eye relative to the other [10,11]. Benign paroxysmal positional vertigo (BPPV) is a vestibular labyrinth disorder that causes short, intense bouts of vertigo with certain head positions. The official pathophysiological cause of BPPV is the movement of otoliths from the utricle's macula through one or more semicircular canals [12]. When the head is positioned so that gravity induces persistent endolymph movement within the affected semicircular canal, BPPV symptoms are triggered. The presence of otoliths within or near the canal or cupula causes subjective vertigo and nystagmus in the plane of the affected canal [13]. Patients with ON who receive physical therapy benefit from improved ocular muscle strength and gaze stabilization. Therapeutic approaches for eye movement abnormalities include vision recovery therapy, neuro-eye therapy, eye mobility training, and compensatory head position activities [14]. This case study presents a typical case of optic nerve inflammation accompanied by strabismus, in addition to the patient's recovery through a highly planned rehabilitation strategy.

## Case presentation

Patient information

A 14-year-old boy visited the neurology outpatient department (OPD) complaining of blurring of vision of bilateral eyes along with dizziness and head rolling while returning to school for 10 days. The bilateral blurring of vision was sudden in onset and gradually progressive. Other associated complaints were weakness in the right upper limb for two days. Medical history revealed that the patient had recovered from typhoid three months ago. Clinical examination and investigation revealed hyperintensity on short-tau inversion recovery (STIR) with mild contrast enhancement in the posterior aspects of bilateral optic nerves (intracranial part) with extension till the optic chiasm in ON. Then, the patient was referred to neurophysiotherapy for further management.

Clinical findings

The patient cooperated and was aware of time, place, and person, and his words, language, intelligence, and memory all remained intact. Visual acuity was 14/24 and 6/12. Ishihara's colored vision revealed 6/17 oculus dexter (OD) and 8/17 oculus sinister (OS). The patient also had a strabismus on the right eye. Snellen chart told 22/32. Further, muscle strength was reduced on the right upper limb (3/5). Reflexes were normal.

Timeline of events

The complete incident's timeframe is depicted in Table 1.

**Table 1:** Timeline of events AVBRH: Acharya Vinoba Bhave Rural Hospital

Incidents	Occurrence
Diminution of both eyes	10/10/2023
Weakness in the right upper limb	16/10/2023
Date of admission in AVBRH	18/10/2023
The date of physiotherapy started	18/10/2023

Diagnostic assessment

**Figure 1:**
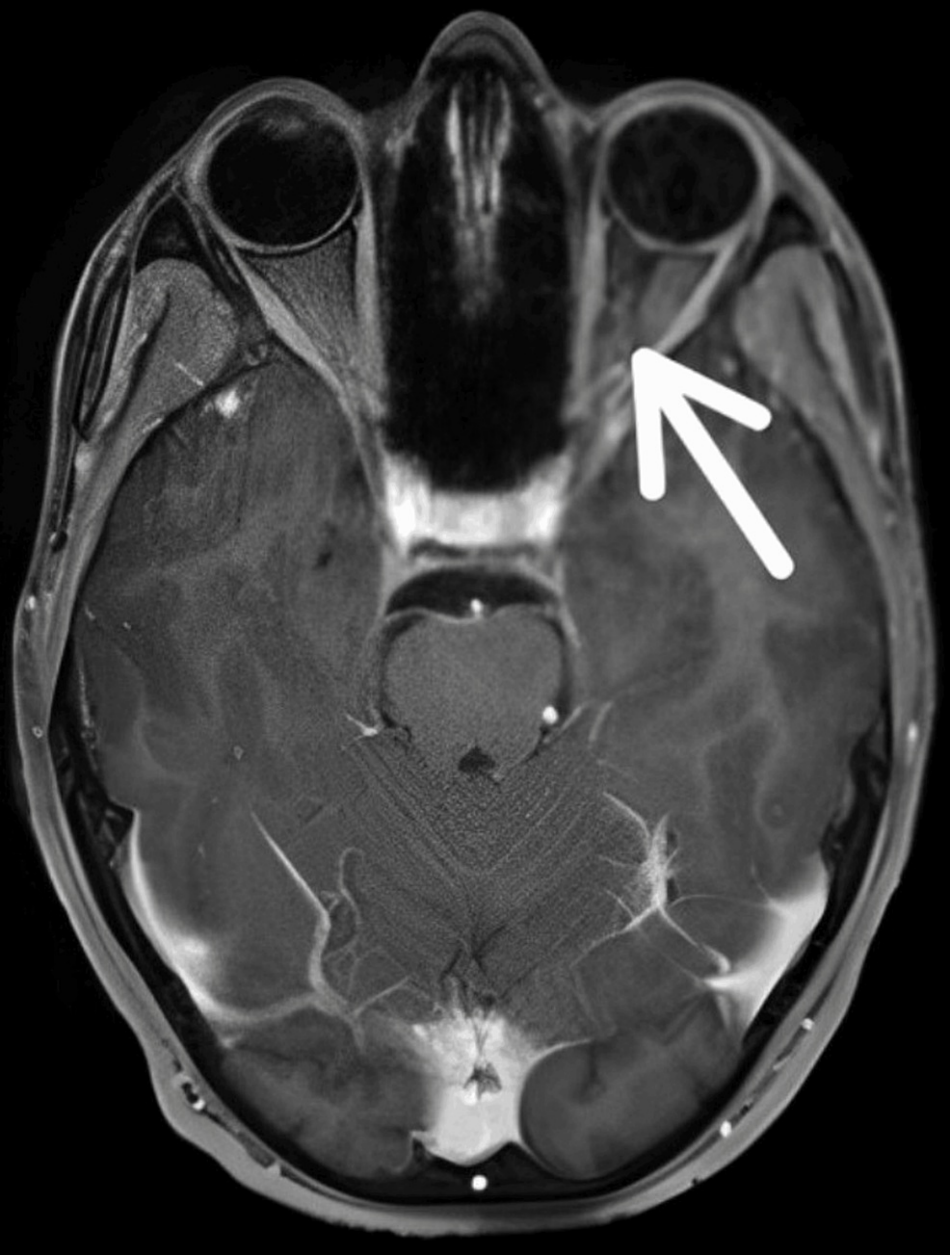
MRI of the brain Arrow indicates hyperintensity on STIR with mild contrast enhancement in the posterior aspects of bilateral optic nerves (intracranial part) with extension till the optic chiasm in optic neuritis MRI: magnetic resonance imaging; STIR: short-tau inversion recovery

Magnetic resonance imaging (MRI) reveals hyperintensity on STIR with mild contrast enhancement in the posterior aspects of bilateral optic nerves (intracranial part) with extension till the optic chiasm in ON shown in Figure 1.

Therapeutic intervention

The goal-oriented physiotherapy intervention protocol included exercises for gaze stability, exercises to strengthen extraocular muscles, habituation exercises, Cawthorne-Cooksey exercises, and exercises to increase visual feedback. All these exercises helped our patient to increase his coordination and further improve his quality of life. The physiotherapy intervention is described in Table 2.

**Table 2:** Goal-oriented physiotherapy intervention

Sr. no.	Goals	Therapeutic intervention	Treatment regimen
1	Patient education	A patient and his relatives are informed about the condition and well-planned physiotherapy rehabilitation	Relatives were educated about the treatment and its importance
2	To strengthen vestibular-ocular reflex	Gaze stability exercises	Ten repetitions of a single set two times each day
3	To increase synchronization among eye and hand motions	Eye-hand coordination exercises	Ten repetitions of a single set two times each day
4	To enhance ocular muscle strength	Eyeball resistance exercises	Ten repetitions of a single set two times each day
5	To enhance visual feedback	Habituation exercises	Ten repetitions of a single set two times each day
6	To enhance coordination	Cawthorne-Cooksey exercises	Ten repetitions of a single set two times each day
7	To improve strength in the right upper limb	Strengthening exercises with 1 litre water bottle	Ten repetitions of a single set two times each day

Outcome measures

**Figure 2:**
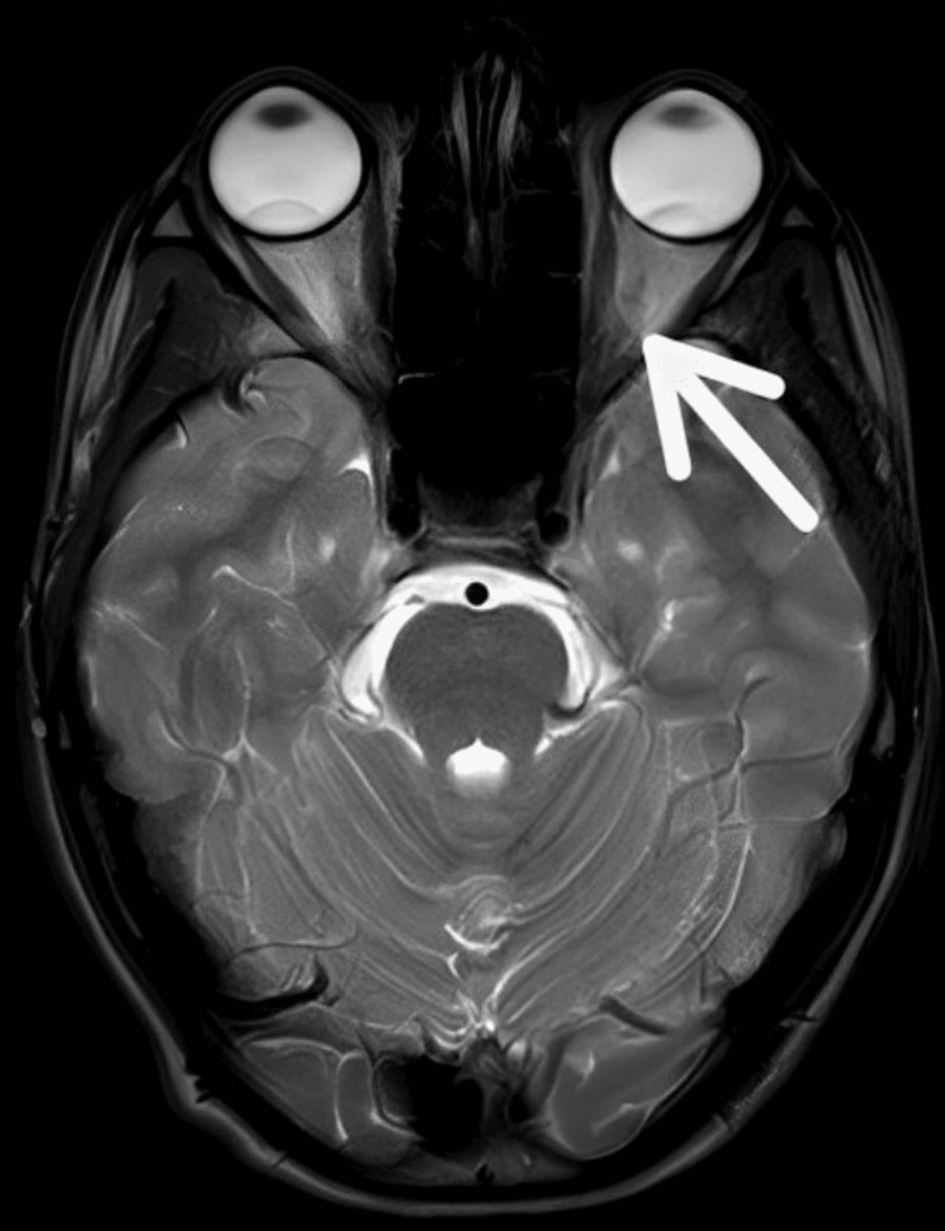
Post-rehabilitation MRI MRI: magnetic resonance imaging

At the end of our rehabilitation, our patient showed significant improvement in the Functional Independence Measure and National Eye Institute Visual Functioning Questionnaire-25. Our rehabilitation consisted of six weeks. At the end of six weeks, physiotherapy measures were taken. Table 3 and Figure 2 show pre- and post-physiotherapy outcome measures and post-rehabilitation MRI.

**Table 3:** Pre- and post-physiotherapy outcome measures

Outcome measures	Pre-physiotherapy intervention	Post-physiotherapy intervention
Functional Independence Measure	50	110
National Eye Institute Visual Functioning Questionnaire-25	40	90

## Discussion

The typical presentation of ON is subacute monocular loss of vision accompanied by discomfort while moving the eyes. Typically, hours or days pass before visual impairment manifests [15]. The majority of patients complain of fogging or blurring of vision. The intensity varies greatly and usually reaches its lowest point in two weeks. According to the results of one study, red-green defects were more probable to be linked with larger foveal area depression than blue-yellow abnormalities; however, the opposite was observed in individuals whose visual fields were dominated by perifoveal abnormalities [16,17].

Our study worked to investigate the impact of visual scanning exercises, task-specific training, and placebo eye workouts on performing everyday tasks and equilibrium in stroke patients having eye movement problems. By improving the precision and the rate at which the affected side's eyes move, the patients were encouraged to learn how to deal with their challenges through visual scanning tasks. The goal of visual scanning activities was to motivate patients to study in order to get over their difficulties, and they need to shift their eyes across the afflicted region more quickly and accurately [18]. The therapy included various interventions like gaze stability exercises, exercises to strengthen muscles of the ocular part, various habituation exercises to improve visual feedback, and Cawthorne-Cooksey exercises, which helped to enhance our patient's coordination.

Hence, we believe that individuals with ON, along with those having inflammatory myopathies and neurological conditions, can benefit from the safe and effective use of a rehabilitation programme under close supervision. BPPV causes far more paediatric dizziness than originally assumed. It is most commonly associated with vestibular headache syndromes and concussion. Paediatric patients are more likely than adults to be involved in lateral, anterior, and numerous canals. Treatment resistance may be exacerbated by vitamin D insufficiency, which has been identified as a risk component for both medication-resistant and recurring BPPV in adults [19,20].

## Conclusions

This case report paves the way for future rehabilitation for patients with ON, and it adds to relevance for rehabilitating patients with ON. It aimed to discuss pathophysiology, the definition of rare pathologic diseases, and the integrative role of physiotherapy in managing such diseases. Based on this report, early integrative neurophysiotherapy can be an effective intervention for managing symptoms of neuromyelitis optica. At the end of our rehabilitation, our patient showed improvement in clinical symptoms and outcome measures. Physiotherapy helps people maximize their residual vision by focusing on areas such as visual scanning, spatial awareness, and eye movement control. This promotes independence and decreases the burden of ON on everyday activities.
